# The complete chloroplast genome sequence of *Michelia compressa*

**DOI:** 10.1080/23802359.2020.1788434

**Published:** 2020-09-01

**Authors:** Dongqin Zhou, Shipeng Lu, Zhaoqi Hou, Jinping Yu

**Affiliations:** Institute of Botany, Jiangsu Province and Chinese Academy of Sciences, Nanjing, China

**Keywords:** *Michelia compressa*, chloroplast genome, sequencing, gene

## Abstract

*Michelia compressa* is an evergreen ornamental tree species. The high-throughput sequencing technology was used to sequence and assemble the chloroplast genome of *Michelia compressa*. Results showed that the chloroplast genome is 160,061 bp in length, of which the inverted repeats sequence (IRs) is 26,581 bp, the large single-copy region (LSC) and the small single copy region (SSC) are 88,097 bp and 18,802 bp, respectively. The GC content of the plastome was 39.2%, with 43.2%, 37.9% and 34.2% in IRs, LSC and SSC, respectively. A total of 132 genes are annotated, 86 protein-coding genes, 37 tRNA genes, and 8 rRNA genes. This study enriched the *Michelia compressa* genomic information which provides the basis for rational exploitation and utilization of germplasm resources.

Magnoliaceae is one of the primitive groups of angiosperms which contains 16 genera that more than 300 plants. Among that *Michelia* as the second largest genus of the family magnoliaceae, consists of about 50 species (Keng 1996), and there are 38 species in China, living mainly from southwest to southeast. The flower buds of this plant are always used as traditional Chinese medicine, which have been used for treating laryngitis, rhinitis, inflammation. But, at present, there are still great differences in the division of the position of the magnoliaceae, especially the subspecies of the family. Chloroplast has been a valuable tool to be used for phylogenetic studies due to its gene conservation and the lack of recombination (Ravi et al. 2008; Lin et al. 2012). Here, we reported the complete Chloroplast Genome of *Michelia compressa*, which provide valuable information for molecular breeding, genetic modification, and phylogenetic study in Magnoliaceae.

The sample of *M. compressa* was collected from Nanjing Botanical Garden MEM. Sun Yat-Sen, Jiangsu, China (32°02′N, 118°28′E). The voucher specimens were stored in the Institute of Botany, Chinese Academy of Sciences, Jiangsu Province (voucher No.320102190105002LY). Total genomic DNA was extracted from the fresh mature leaves of *M. compressa* and sequenced on an Illumina Hiseq Xten platform (San Diego, CA). Genome sequences were screened out and assembled with Novoplasty v 2.7.2 (Dierckxsens et al. 2017).

The sequence of *M. compressa* genome has been deposited in Genbank (accession number: MN604380). The total length of *M. compressa* chloroplast genome was determined to be 160,061 bp in length, of which the inverted repeats sequence (IRs) is 26,581 bp, the large single-copy region(LSC) and the small single copy region (SSC) are 88,097 bp and 18,802 bp, respectively. The GC content of the plastome was 39.2%, with 43.2%, 37.9% and 34.2% in IRs, LSC and SSC, respectively. The genome is structured with 132 genes, including 86 coding protein genes, 37 tRNA genes and 8 rRNA genes. Among these genes, 18 genes were repeated IR regions, including 7 protein-coding genes, 7 tRNA genes and 4 rRNA genes.

A molecular phylogenetic tree was constructed with MEGA 6.0 (Tamura et al. 2013) based on the maximum likelihood method using a dataset of the complete genome sequence of *M. compressa* with those of 14 individuals of Magnoliaceae obtained from Genbank. The result revealed that *Michelia* was a polyphyletic genus. *M. compressa* was closely related with *Mangolia laevifolia* and *Michelia odora*, and nested within the other Magnolia species (Figure 1).

**Figure 1.**
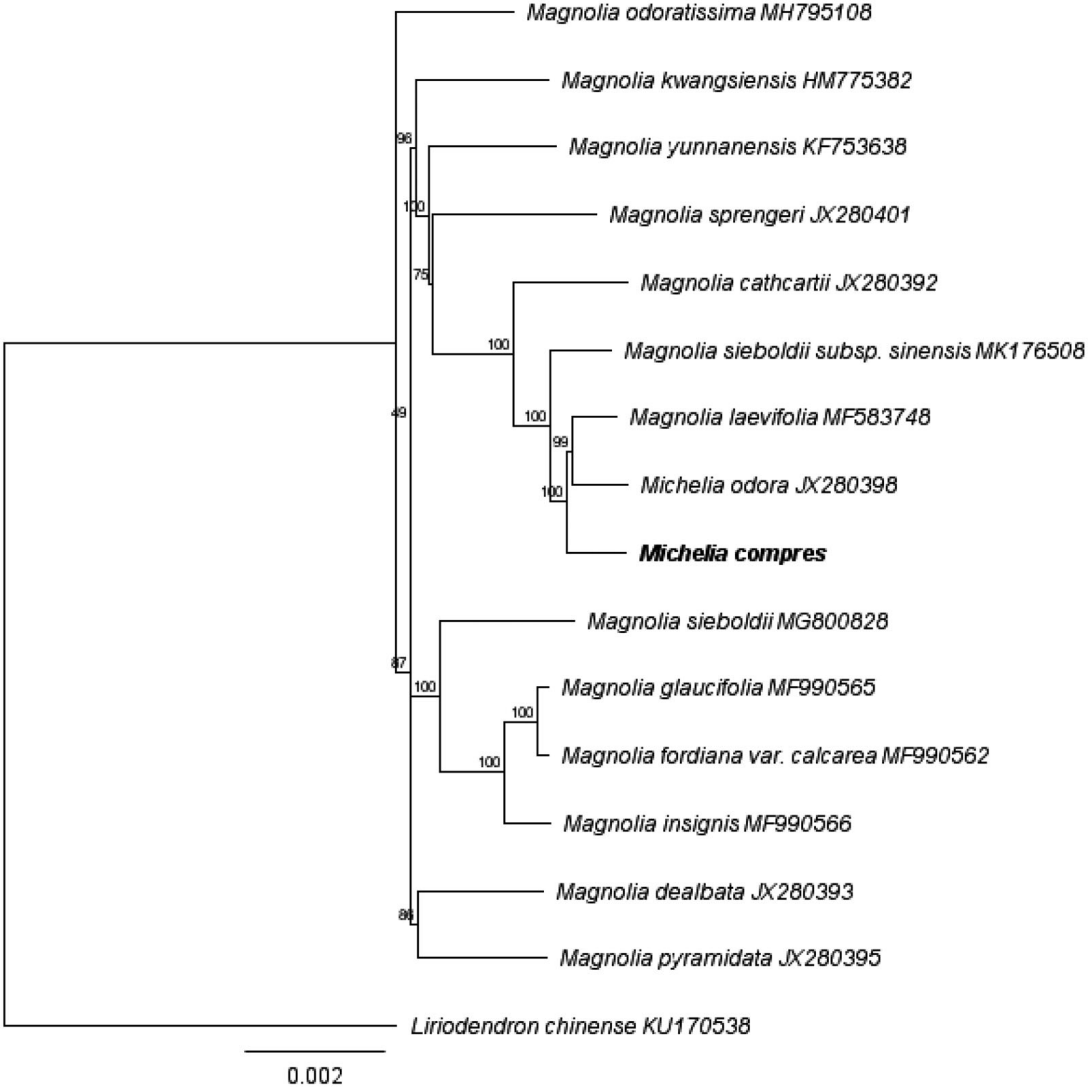
Maximum likelihood phylogenetic tree based on the complete genome sequences of *Michelia compressa* with those of 14 individuals of Magnoliaceae.

## Data Availability

The data that support the findings of this study are openly available in GenBank (https://www.ncbi.nlm.nih.gov) with the accession number is MN604380.
